# Antibiotic Resistance in Syria: A Local Problem Turns Into a Global Threat

**DOI:** 10.3389/fpubh.2018.00212

**Published:** 2018-08-02

**Authors:** Mihajlo Jakovljevic, Sanaa Al ahdab, Milena Jurisevic, Sulaiman Mouselli

**Affiliations:** ^1^Global Health, Economics and Policy, Faculty of Medical Sciences, University of Kragujevac, Kragujevac, Serbia; ^2^Faculty of Pharmacy, Arab International University, Daraa, Syria; ^3^Department of Pharmacy, Faculty of Medical Sciences, University of Kragujevac, Kragujevac, Serbia; ^4^Faculty of Business Administration, Arab International University, Daraa, Syria

**Keywords:** Syria, pharmaceuticals, market, antibiotics, resistance, bacteria, civil war, crisis

## Abstract

Pharmaceutical sector of Syrian Arab Republic before the war was characterized by bold and successful development since the late 1980s. With the beginning of war in the country back in March 2011, momentum has changed significantly. Traumatism, communicable diseases related to morbidity and mortality as well as wound infections became particularly hot public health concern. This relates not only to the direct victims of military conflict but also to the displaced civilians, refugees, and ordinary citizens alike. Evolving legislative framework in Syria since 1980s tolerated dispensing of antibiotics without appropriate prescription. Such practice led to spreading of antibiotic resistance among the local bacteria frequently causing both community-acquired and nosocomial infections. Laboratory findings of resistant bacteria strains among the Syrian refugees in some European countries serve as evidence of concern spreading far beyond Middle East. Practice of self-diagnosis and self-medication with antibiotics by patients themselves and restraint to pharmacist advice is widespread. A number of recommendations is presented to stakeholders to compact antibiotic resistance after the peace is established in the country. The successful implementation of such recommendations is the way to preserve shrinking golden reserve of highly potent antibiotics as it is the last defense line against resistant bacterial strains causing severe life—threatening infections.

## Humanitarian crisis, war, and pharmaceutical market—legacy of Syria

Pharmaceutical markets worldwide exhibit great diversity in terms of prescription and dispensing patterns and value-based turn over (1). Their dynamics is grounded in the legacy of health care system establishment. Traditional medical services provision and financing differ profoundly from one global region to another. Few core examples of such diversity are major historical systems ranging from British Beveridge (2) to Soviet Semashko (3) and from German Bismarck (4) to Chinese contemporary health system (5) war.

Middle East presents a distinctively different region of the Old World (6). Ninety year old historical records on practice of pharmacy in Syria describe it as a former Ottoman province under French mandate. Its humble market presence of seldom drugs recognized in official clinical medicine of post WWI era was following Turkish traditions largely (7). The aim of this article is to review the current state of antibiotic use in Syria and clarify the main reasons behind the widespread irrational use of antibiotics in order to suggest venues of interventions.

In Syria, the main source of health financing comes from the government's budget presented by the Ministry of Health and other ministries such as the Ministry of Higher Education, the Ministry of Defense, and the Ministry of Local Administration (8). Public health services in places such as health centers and hospitals have long been offered free of charge. However, since 1998, patients have had to pay little charges to get access to certain health services in some public hospitals (9). Although inexpensive, people more often utilize these facilities in certain medical conditions, such as complicated surgeries, hemodialysis, cancer chemotherapy, and blood disorders.

Public health services in Syria are commonly of insufficient quantity and quality (State planning organization, 2006). Hence, patients have to visit private clinics, where they make out-of-pocket payments. Those payments slightly increased from 59.6% in 2000 to 61% in 2008 (10). No national insurance system that covers all population exists in Syria. Some small-scale health insurance schemes offered limited coverage to individuals in certain public companies, ministries and professional associations (11). Since 2004, private insurance companies have provided limited services to individuals (8). The high out-of-pocket spending and the absence of a national health insurance system drove patients to pharmacists to dispense medicines.

Before the current war began in 2011, Syria was recognized among the Arab League nations for its strong domestic pharmaceutical industry (12). Back in 1988, it had sufficient supply of educated clinical physicians. Occasional drug shortages and lack of access to vital medicines were recognized as the core weakness of the national health system (13). Since the late 1980s until the late 2000s, governmental supported this sector to cover almost 90% of national needs compared to only 6% in the beginning (14). Notable $150 million valuable annual exports were achieved toward few dozen of Organization of Islamic Conference (OIC) countries. Local labor market, heavily dominated by women consisted of over 17,000 employees and even 54 local pharmaceutical factories (14).

Coming back to contemporary momentum, we witness a disastrous war inside the Syrian Arab Republic as a consequence of complex chain of events following the Arab Spring colored revolutions (15). As in several similar previous large conflict areas such as Somalia, Afghanistan, the Democratic Republic of Congo and Haiti, health care provision, and outcomes are greatly affected (16). Relief in severely disrupted countries is achieved largely by multilateral donor agencies such as the Red Cross (17), WHO Division of Emergency and Humanitarian Action, Red Crescent (18) and many others (19). With the intent to provide more equitable and just outreach of essential drugs supplies toward most vulnerable citizens, some of the UN agencies such as WHO even created guidelines for distribution of donation medicines aimed to cover drug shortages (20). Probably, the most notable example of health system crisis in the surrounding Middle Eastern nations are reports coming from Iraq described as early as of 2003 (21).

Syria is no exception to similar vulnerabilities. Threatened supplies of essential medicines is currently the case even far outside major refugee migration routes (22) and war torn areas of the country. Major multilateral agencies such as the Médecins Sans Frontières (MSF) have claimed serious degree of disrupted access to basic health care for the ordinary citizens. Official WHO estimate was that Syria needed a total of $900 million worth of essential medicines and supplies in a single year following March 2013. However, keeping in mind the international financial climate at that time and the stage of the war, major donors only partially covered the urgent needs. Consequences were particularly striking in some clinical areas such as diabetes, cancer care, appropriate blood storage and testing facilities necessary for safe transfusions in surgery (22).

## Concerning growth of antibiotic resistance in Syria

If we think about the nature of modern urban warfare, we could notice a long term trend that infantry weaponry is actually being made with the purpose to make wounds instead of killing at the first place. This trend in military equipment manufacturing is purely related to industrial and strategic reasons (23). This sad truth had profound and disastrous consequences both for the combatants (military personnel) of all fractions and civilians in Syria. The huge frequency and scale of traumatism impose a burden of appropriate blood transfusion provision and need to cure pyogenic wound infections. Bacterial causes are primarily aerobic Streptococcus, Staphylococcus species, and anaerobic Clostridium bacteria, notorious for causing gas gangrene.

Here we face another core issue even when common broad-spectrum antibiotics are at disposal of major hospitals and day care centers throughout the country. Antibiotic resistance presents an alarming threat to antimicrobial therapy. This occurring public health concern extends far beyond Syria toward other Middle Eastern neighborhood countries and the European countries alongside major refugee evacuation routes (24). The roots of this problem are inherited in the Syrian health system. The epidemiological burden of infections morbidity and mortality continues to grow further (25) as documented in the framework of Global Burden of Disease Project (26). Workload for the local and international health workforce and costs of care are largely attributable to traumatism, community-born and nosocomial bacterial infections arising from neglected chronic conditions (27). These refer to poverty and absence of decent medical care and access to medicines as indirect consequences of war. Contributions to release the suffering and medical expenditure are paid by Middle Eastern and high-income donor countries worldwide, given the wide spread of Syrian refugee crisis (28). The evidence clearly suggests that together with migration of patients with infections, bacterial resistance also moves (29). This study looks into the evidence compiled from samples collected in Syria, Jordan, and Europe and the reasons behind this problem (30).

A study on the Syrian antibiotic resistance performed by Omran and Askar at Al-Mouwasat University Hospital (31) demonstrated a decline in the bacterial resistance against the antibiotics that were included in the study in comparison with earlier studies carried out at the same hospital (30). Antibiotic resistance may develop in weeks, months, or over a period of years. The increase in travel from Syria to different parts of the world due to the War indicates that the antibiotic resistant microbes can be transported within hours or days to other locations. A report from a charitable hospital in a neighboring country, Jordan, has documented cases of clinical failure to the first-line choice for prophylaxis and treatment of skin and soft-tissue infections (narrow-spectrum cephalosporin) (32).

In 2016, 48 Syrian migrants arrived in Italy. Upon their arrival, they received a physical examination and were subject to microbiological surveillance by blood, rectal, pharyngeal, and nasal swabs collection. Swabs were delivered and examined in local Italian clinical pathology and microbiology laboratory. Pathological analysis showed that all the 48 migrants were negative for HBV, HCV, and HIV infections. However, a large number of unusual gram-negative bacteria species were isolated and among the isolates, different strains resistant to antibiotics were found (33). European centers (for healthcare of asylum seekers) also reported multi-drug resistant (MDR) pathogens among wounded adult patients and refugees from Syria. In Germany, among refugees from Syria in 2016, the rate of colonization with gram-negative MDR pathogens was 60% (34).

In Syria, patients are frequently self-diagnosed and self-medicated, or they seek the advice of their local pharmacists (35) with prevalence rate of 57% (36). Over-the-counter sales of antibiotics have been reported in many countries of the Middle East; the prevalence rate of antibiotic self-medication ranged from 19 to 82% (37). Pharmacists, who have to be acquainted with adverse effects of antibiotics misuse, provide antibiotics over the counter without prescription fearing that their customers would go elsewhere (35). The supply of an antibiotic from a pharmacy without a prescription usually involves a consultation with a pharmacist. In previously published study, one out four participated pharmacists in Syria considers him/herself qualified to give the right medicine (38). Also, they reviled that among chosen pharmacies, 13.8% of pharmacies are working without a pharmacist. This fact is obviously leading to providing misinformation about drugs and selling antibiotics according to popular demand (39). The result of this action is that citizens acquire antibiotic without proper diagnosis and are at higher risk of developing antibiotic resistance.

It is very easy to purchase antibiotics in Syria without prescription (35). A cross-sectional study carried out on pharmacists in the capital, Damascus, found that 87% of them sold antibiotics without prescription, 10% accepted with prescription, and only 3% refused to give antibiotics without prescription (38). Pharmacists included in this study treated recurrent simple infection with common antibiotic, such as amoxicillin, with or without clauvalenate or cephalexin. A similar study conducted in Aleppo also showed that the overall prevalence of antibiotic drug dispensing without prescription was 85.5% (39).

Over long time, in a loose regulatory setting, physicians have frequently mistakenly prescribed antibiotics as a cure to diverse communicable diseases, such as flu and common cold. It is well-known that viruses are the origin of these diseases, therefore antibiotics are ineffective (40–42). Antibiotic sensitivity patterns are rarely checked. Doctors prescribe antibiotic as soon as possible in a fake attempt to save the patient's time and money. They sometimes even prescribe high doses of wide spectrum antibiotic to show patient families their ability to improve the clinical outcomes in a short time. Tendency of physicians to ignore good clinical practice guidelines is to a lesser extent evident even in high-income European, Asian, and North American clinical and academic milieu (36). However, in the Middle East, it has far more concerning extent (43).

Patients, on the other hand, who may not be aware of the side effects of such antibiotic treatment, may misuse their prescribed antibiotic by stopping the course of treatment too early, when the painful symptoms begin to relieve (44). They may also reuse the same antibiotic drug when they have similar symptoms after a period of time. This sort of poor patient compliance has been documented across a variety of low and middle-income countries even in full social peace and welfare living (37, 45).

In 2010, a cross-sectional study was carried out on 430 randomly selected adult residents of Kalamoon in Syria using standardized questionnaire. The study found that 85% had taken antibiotic medicines in the past 4 weeks and 34% were not aware of the adverse effects of antibiotics. Only 43% (out of the 85%) were prescribed the antibiotic by a physician to treat the condition, while 57% used an old prescription or took someone else's advice. This clearly indicates that the laws that control purchasing of antibiotics are ignored (46).

It is well-known that Syria still has the largest number of pharmaceutical companies compared to most other Arab countries. Although this branch of the economy suffered heavily due to military actions, domestic companies, despite war conditions, are capable to provide antibiotics at reasonable prices. Although antibiotics are not cheap, they are affordable to many middle-income households and patients.

Syria has a national-level committee designed to address antibiotic treatment related issues, including resistance. However, it has insufficient funding, resources, and leadership and thus it cannot play a significant role in controlling prescription, dispensing, and sale patterns. Moreover, Syria does not have a national policy restricting the availability of antibiotic medicines without a prescription (47).

In Syria, three national authorities deal with antibiotic resistance: The Central Infection Prevention and Control Committee, The Directorate of Drug Affairs, and The Department of Infection Control in Hospitals' Directorates. Despite of the presence of those national bodies, the WHO officer reported that the priority given to antimicrobial resistance had been declining, due to the current war. Syria has also national laboratories with the ability to identify resistant bacteria; however, these laboratories do not produce reports or have a monitoring or reporting system for antibiotic resistance. Moreover, Syria does not participate in the regional infection control network (47).

The Ministry of Health in Syria, three decades ago, passed a law (Number 2/T, dated 12/1/1988) that determined drugs that could be sold to people without a medical prescription and antibiotics were not included in the list of drugs [Syrian Syndicate for Pharmacists—Laws and orders that coordinate pharmacy career in Syria]. Damascus, Syrian Syndicate for Pharmacists, 1994 [In Arabic]). Another law (Number 2/T, dated 23/1/1992) prevented pharmacists from reselling prescribed antibiotic to the same individual without the permission of a physician and prevented physicians from prescribing an antibiotic more than twice to treat the same infection for the same individual (Syrian Syndicate for Pharmacists, 1994). However, those regulations are not clearly stated or strictly enforced (48).

## Learning from the Syrian's experience

Despite the global interest of the consequences of AMR, there is no sense of urgency about the current AMR status in Syria. Antimicrobial resistanceis not anymore purely a national concern. It turns to be an international issue with financial consequences. Hence, efforts should be coordinated in a Syrian national strategic plan to control the development of AMR. This can be done through reactivating the role of existed national committee and imposing more restrictions on dispensing antibiotics without prescriptions. Any savings made from the reinforcement of prevention and control activities are cost-effective and financial deficit should not be a barrier.

The establishment of antimicrobial surveillance system in Syria will be a good start. This surveillance system could benefit from the instructions of European Antimicrobial Resistance Surveillance Network (EARS-Net) reporting protocol (49) similar to the one suggested in Italy (50). The aim of such system would be to produce a reliable data on the sales of antibiotics from pharmacies as well as the development of AMR from laboratories. Accumulated data can be made publicly available on the Ministry of Health website with regular periodic updates to track and monitor the progress of AMR.

Given that the high level of AMR is the result of purchasing antibiotics without prescription (35, 38), it is necessary to increase the awareness of current and possibly future pharmacists of the negative consequences of AMR. Educational programs among community pharmacists and pharmacy students may help enhance the rational use of antibiotics with similar programs been suggested in other countries (51). Similar awareness programs among physicians may also address this concern. The second reason for the widespread irrational use of antibiotics is the soft enforcement of legislations regarding the illegal dispensing of antibiotics. Strong enforcement of those legislations includes imposing fines on the inappropriate dispensing as seen in the Republic of Srpska (52) or temporarily suspending pharmacists' licenses, which may reduce the illegal selling of antibiotics. Another action could be taken by the Ministry of Higher Education through designing teaching modules, where pharmacy students are taught to be health educators, and incorporate respecting legislations in their code of Ethics. Syrian Syndicate of Pharmacists should also play a role in promoting FIP and WHO guidelines of dispensing antibiotics through continuous education to pharmacists especially those located in mid and low educated areas (53, 54). In 2017, WHO reported that three pharmacy graduates, in collaboration with Syrian Syndicate of Pharmacists, started a campaign to inform pharmacists of their role in preventing antibiotic resistance (55). They reached over 400 pharmacies in Damascus in addition to healthcare centers and hospitals. This may be a promising strategy to reduce antibiotic resistance.

We also recommend activating the role of the national committee for the rational use of antibiotics to play its expected role as a national coordinating body responsible for enhancing the prudent use of antibiotics, similar to other countries (56, 57). Stakeholders can also reduce irrational antibiotic use by expanding health insurance coverage; this will encourage patients to visit physicians, rather than pharmacists. Hence, only physicians can make decision whether it is necessary to take antibiotics.

The negative economic impact of AMR involves increasing mortality rate and permanently reducing the size of population and prolonging the periods of sickness and, consequently that could reduce the labor workforce efficiency. A study by Taylor estimates the GDP loss due to AMR in the MENA countries (including Syria) to range between USD 2 billions and 159 billions per year over 40 years (58). Such large costs impose additional burden to the already exhausted Syrian economy recovering from the costly crisis (59). The implementation of the above mentioned recommendations shall contribute toward building up Syrian welfare state and a decently efficient and cost-effective health system once again in the near future.

## Author contributions

MJa, SA, MJu, and SM all contributed equally to the acquisition of published evidence, selection, and screening of evidence for its validity and methodological quality. All three authors have revised and contributed significantly to final manuscript for important intellectual content fulfilling all ICMJE conditions for authorship.

### Conflict of interest statement

The authors declare that the research was conducted in the absence of any commercial or financial relationships that could be construed as a potential conflict of interest.
